# Determinants of short birth interval among women in South Gondar, Ethiopia: community-based unmatched case-control study

**DOI:** 10.1186/s13690-021-00567-7

**Published:** 2021-04-09

**Authors:** Gedefaye Nibret Mihretie, Fentahun Yenealem Beyene, Bekalu Getnet Kassa, Alemu Degu Ayele, Tewachew Muche Liyeh, Binyam Minuye Birihane

**Affiliations:** 1 Department of Midwifery, College of Health Sciences, Debre Tabor University, Debre Tabor Town, Ethiopia; 2 Department of Midwifery, College of Health Sciences, Bahir Dar University, Bahir Dar Town, Ethiopia; 3 Department of Pediatric Nursing, College of Health Sciences, Debre Tabor University, Debre Tabor Town, Ethiopia

**Keywords:** Institution birth, Rural district, Short birth interval, Reproductive age women

## Abstract

**Background:**

The effect of short birth interval on socio-economic, negative maternal and child health outcomes remains common in developing countries. This study aimed to assess determinants of short birth interval among reproductive age women, who gave birth in health institution for last six-month in South Gondar, Ethiopia 2019.

**Methods:**

Community-based unmatched case control study design was conducted from February 1 to March 30, 2019. Sample size of 150 was included by simple random sampling technique. The data was collected by semi-structured and pre-tested face to face interviewer-administered questionnaire from selected respondent. The collected data was entered with Epi-Data version 3.1 and analyzed by using SPSS version 23 software. Bivariate and multivariable analyses were used to examine the association. Odds ratio, 95% CI and *P*-value < 0.05 were used to determine the statistical association.

**Result:**

The mean age of the respondents was 32.42 (SD ± 5.14) and 35.12 (SD ± 5.86) for cases and controls, respectively. Mothers not used contraceptives (AOR = 6.29, 95% CI (1.95, 20.24)), participants who had ≤2 alive children (AOR = 5.57, 95% CI (1.47, 21.13)), mothers who breast fed less than 24 months (AOR = 3.42, 95% CI (1.38, 8.46)), husband decision on contraceptives utilization (AOR = 2.69,95% CI (1.05,6.88)) and mothers who did not have history of antenatal care follow up (AOR = 3.52, 95% CI (1.27, 9.75)) were associated with short birth interval.

**Conclusion:**

The optimum birth spacing plays a vital role in decreasing fertility and the morbidity and mortality of mothers and children. Thus, providing health information on the benefit of breast feeding, follow-up of antenatal care during pregnancy, use of contraceptives after delivery and encouraging mothers to make decisions about their own health and use of contraceptives to optimize birth spacing for rural communities.

## Background

Short birth interval is defined as an interval between two consecutive births less than 33 months (birth to birth interval). After a live birth, the recommended interval before attempting the next pregnancy is at least 33 months in order to reduce the risk of adverse maternal, perinatal and infant outcomes [1]. Institutional delivery means giving birth to a child in well-equipped medical institution under the overall supervision of trained and competent health personnel where there are more facilities available to handle the situation and save the life of the mother and child [2]. Birth interval is an important determinant of the rates of population growth and socio-economic status of communities and it offers a great potential in protecting the health status of the mothers, and improving outcome of subsequent pregnancy [3].

More than 200 million women in developing countries want to either space or limit pregnancies and still lack access to options for family planning [4]. Demographic Health Survey (DHS) studies show a high level of short birth intervals in many African countries (Rwanda: 20%; Uganda: 25.3%; Ethiopia: 20.4% and Cameroon: 21.3%) [5]. Ethiopia has experienced a significant number of infant and neonatal mortality compared to the overall average rate of infant and neonatal mortality reported in Africa [6]. Globally, studies showed that the probability of maternal and child death increase greatly as inter birth intervals fall below 36 months [7]. Short birth intervals highly associated with adverse health outcomes, including infant, child and maternal mortality and mortality, preterm birth and low birth weight [8, 9].

Studies in Tanzania showed that short birth interval women will not have sufficient time to recover in terms of socio-economic, cultural, psychological and physical body preparedness for the subsequent pregnancy and failure of vaginal birth after caesarian section [10]. There were different factors associated with birth interval. Young maternal age [11], women who had no formal education [12], women with secondary education or higher and Christian women were more likely to experience a short birth interval [13]. Study in Pakistan indicated that preceding child sex (female) has statistical association with short birth interval [11]. However, evidence from Ethiopia demography and health survey (EDHS) 2011 showed that sex of the child has no statistically significant association with birth interval [11]. Other studies indicated that mothers who had previous stillbirth were more likely to have long birth intervals [13–15].

According to the study conducted in Manipur, Pakistan, Nigeria and Mekele duration of breast-feeding was found to be an independent predictor of birth intervals. It showed that women who breast fed their child for longer than 24 months were more likely to have longer birth intervals [14–17]. As opposed to the above, finding from Lemo District in, showed that duration of breast-feeding has inverse relationship with birth intervals [18]. Parity in Manipur indicated that birth interval increases as parity increase [15]. Contraceptive utilization was also mentioned as one of the major determinants of length of birth intervals. Women who used modern contraception were more likely to have longer birth intervals than those who never used any contraceptive method [17].

Even though, institutional delivery service utilization is one of the key and proven interventions to reduce maternal and child morbidity and mortality, short birth interval remains a major challenge in developing countries including Ethiopia particularly rural community [19]. And shorter birth intervals were more common among rural women in Ethiopia [20]. Therefore, assessing determinants of short birth interval among reproductive age women are very vital in designing, implementing and monitoring effective intervention programs for these particular group.

## Methods and material 

### Study settings

Community-based unmatched case control study design was employed from February 1 to March 30, 2019 in rural Farta woreda, South Gondar, Ethiopia. Farta woreda is one of the 15 woredas in South Gondar Zone, located in Amhara National Regional State, Ethiopia. Farta woreda is located 590 km North West from the capital city of Ethiopia, Addis Ababa. The woreda is subdivided in to 32 rural and 1 urban kebeles. In Farta woreda there are 10 health centres, 56 health posts, and 4 privet health clinics. Based on the 2015 demographic survey projection a total of 276,144 populations in the woreda, among these 139,923 are males and 136,221 are females. Women who have less than one-year child is estimated to be 3173.

#### Study design

Community-based unmatched case control study design was employed.

#### Source population

All reproductive age women residing in rural Farta Woreda who experienced at least two consecutive births and the last delivery occurred in the institution and within the past six months prior to the data collection.

#### Study population

All reproductive age women residing in selected kebeles in rural Farta Woreda who experienced at least two consecutive deliveries and the last delivery occurred in the institution and within the past six months prior to the data collection.

#### Cases

Reproductive age women who have history of short birth intervals between the latest two consecutive births.

#### Controls

Reproductive age women who have history of optimal birth intervals between the latest two consecutive births.

### Inclusion criteria

#### Controls

All women who gave alive birth within the last six months and have at least two consecutive births with birth interval of 33 and 59 months between the latest two successive live births and the last delivery was in health institution birth.

#### Cases

All women who gave birth within the last six months and have at least two consecutive alive births with birth interval of less than 33 months between the latest two successive alive births and had history of last institutional birth.

#### Exclusion criteria

Mothers who had history of abortion between the last two consecutive births and women who live less than 6 months in the study area.

### Sample size determination

The sample size was determined by using double population proportion formula for unmatched case control study using Open EPI-Info version 7 software by taking of contraceptive utilization from previous study was chosen as an independent variable, since it gives a maximum sample size. The proportion of mothers who utilize contraceptive among controls (controls exposed) 33.25% and not utilize among cases (cases exposed) were considered to be 60.0% [21]. Accordingly, a 5% level of precision, a power of 80%, and a **1:2** allocation ratio of cases to controls was assumed. An additional non response rate of 10%. Based on the assumptions, the final sample size was determined to be 150 (50 cases and 100 controls).

### Sampling technique procedure

Ten rural kebeles were selected by simple random sampling methods to get the representative sample size. Then, sampling frame was prepared from health extension workers registration book having household identification number. Using respective household identification number, frames of households containing study subjects defined as cases and controls was prepared for each selected kebeles. Then, proportional allocation technique was employed to determine the study participants from each kebele both for cases and controls.

Finally, reproductive age women who had at least two consecutive births and whose last delivery been within the past six months prior to the study was selected using simple random sampling technique from the existing sampling frame by lottery method. Participant who were not present at the time of data collection; three revisits were made to interview the woman. In the case of more than one eligible participant in the household, lottery method was used to select only one.

#### Dependent variable

Short birth interval practice.

#### Independent variables: socio- demographic variables

Age, occupational status of the mother, marital status of the mother, age at first marriage, ethnicity, educational status of the mother, religion of the mother, husband’s educational status, husband’s occupational status,

#### Reproductive health related variables

Age at first pregnancy, gravidity, parity, number of living children, sex of the preceding child, delivery place of the preceding child, survival status of the preceding child, pregnancy plan, antenatal care follows up during previous pregnancy, decision-making power about family planning, maternal and paternal attitude towards contraceptive utilization, breast-feeding practice, type of contraceptive and contraceptive utilization.

### Operational definitions

**Short birth interval**: when the duration between two consecutive births is less than 33 months.

**Optimum birth interval**: when the duration between two successive births is 33 to 59 months.

**Long birth interval**: when the duration between two successive births is greater than 59 months.

**Birth to birth interval** = birth-to-pregnancy interval (24 months) plus nine months (24 + 9 = 33 months) [1].

### Data collection tools and procedures

The data were collected by using interviewer-administered, pretested and semi-structured questionnaires after reviewing previous literatures. The questionnaire was first developed in English and translated to local language (Amharic) then back to English by language experts to keep its consistency. Five health extension workers who were familiar with the local language and custom were recruited as data collectors. Two-degree midwives were assigned as supervisor or for the data collectors and overall supervision also made by the investigators. Training was given for data collectors and supervisors for two days on data collection procedures, interview techniques, and confidentiality of the information obtained from the respondents.

### Data quality assurance

Data quality was ensured during collection, entry and analysis. Before conducting the main study, pretest was carried out on 5% of the sample size outside of the selected kebeles and necessary modification has been made. The principal investigator and supervisor conducted a day-to-day on-site supervision during the whole period of data collection. At the end of each day, the questionnaires were reviewed and checked for completeness and accuracy by the supervisor and investigator and corrective discussion was undertaken with all the research team members.

### Data analysis

After coding, the data was entered using Epi-data version 3.1 and then exported to SPSS version 23 software for analysis. Bivariate analysis was executed to examine crude association of predictors with short birth intervals. Finally, variables having *p*-values ≤0.2 on bivariate analysis were selected as candidates for multivariable analysis and then the independent effect of predictors on short birth interval was determined. Odds Ratio and 95% CI was used to measure the statistical association. *P*-values less than 0.05 were used to determine the statistical significance of the tests. Finally, the results were presented in texts and tables.

### Ethical consideration

After approval, ethical clearance was obtained from Institutional Review Board (IRB) of Medicine and Health sciences, Bahir Dar University. Permission letter was obtained from Farta woreda health office before starting data collection. At the beginning of the data collection, written informed consent was obtained from each respondent after thorough explanation of the purpose and the procedures of the study. Respondents were also informed that all the data obtained from them were kept confidential and anonymous. To ensure confidentiality, names of respondents were replaced by code numbers.

## Results

### Socio-economic characteristics of participants

This study included a total of 150 child bearing age women who had at least two consecutive births with a response rate of 100%. The mean age of the respondents was 32.42 (SD ± 5.14) and 35.12 (SD ± 5.86) for cases and controls, respectively. About 92.0% of the cases and 97.0% of the controls were married. With respect to religion 96.0% cases. Eighty-four percent of the cases and 85.0% of the controls were farmers (Table 1).

**Table 1:** Socio-demographic characteristics of reproductive age mothers (*N* = 150) in Farta Woreda, South Gondar Zone, Ethiopia, 2019

Variables	Case(*n* = 50)		Control(*n* = 100)	
	Frequency	Percent (%)	Frequency	Percent (%)
Maternal age				
21–25	5	10.0	6	6.0
26–30	13	26.0	21	21.0
31–35	17	34.0	19	19.0
36–40	13	26.0	34	34.0
≥ 41	2	4.0	20	20.0
Marital status				
Married	46	92.0	97	97.0
Single/widowed/divorced	4	8.0	3	3.0
Age at first marriage				
12–18	21	42.0	60	60.0
19–24	22	44.0	36	36.0
≥ 25	7	14.0	4	4.0
Religion				
Orthodox	48	96.0	99	99
Muslim/protestant	2	4.0	1	1
Educational level of mothers				
unable able to read and write	15	30.0	37	37.0
able to read and write	18	36.0	36	36.0
grade 1–8	10	20.0	16	16.0
grade 9–12	5	10.0	9	9.0
collage and above	2	4.0	3	3.0
Mothers’ occupation				
house wife	21	42.0	55	55.0
farmer	19	38.0	33	33.0
merchant	7	14.0	7	7.0
government employee	3	6.0	5	5.0
Husbands’ educational level				
unable able to read and write	4	8.0	4	4.0
able to read and write	14	28.0	40	40.0
grade 1–8	20	40.0	39	39.0
grade 9–12	5	10.0	11	11.0
collage and above	3	6.0	4	4.0
Husband’s occupation				
Farmer	24	48.0	82	82.0
Daily laborer	16	32.0	9	9.0
Merchant	6	12.0	6	6.0
Sources of information				
Radio/ TV/ mobile	13	26.0	36	36.0
No source of information	37	74.0	64	64.0

### Reproductive health characteristics of respondents

The mean duration of birth interval was 21.08 with SD ± 5 .30 and 44.84 ± 7.26 months among cases and controls, respectively. Eight percent of the cases and 10 % of the controls had history of still birth before the preceding birth. Majority (62.0%) of the cases and 64.0% of the controls breast fed 13–23 months. Sixty eight percent of the cases and 88 % of the controls utilized modern contraceptive method after the delivery of the preceding child before the last child. Thirty percent of cases and 43.0% of controls had no ANC visits during their last pregnancy (Table 2).

**Table 2:** Obstetric, breast feeding and contraception history of reproductive age mothers (*N* = 150) in Farta Woreda, South Gondar Zone, Ethiopia, 2019

Variables	Case(*n* = 50)		Control(*n* = 100)	
	Frequency	Percentage (%)	Frequency	Percentage (%)
Age at fist pregnancy				
15–19	14	28.0	46	46.0
20–24	27	54.0	44	44.0
> =25	9	18.0	9	10.0
Number of living children				
0–2	16	32.0	22	22.0
3–4	24	48.0	46	46.0
> =5	10	20.0	32	32.0
Abortion before the preceding child				
Yes	6	12.0	24	24.0
No	44	88.0	76	76.0
Still birth before preceding child				
Yes	4	8.0	10	10.0
No	46	92.0	90	90.0
Sex of the preceding child				
Female	18	36.0	59	59.0
Male	32	64.0	41	41.0
Status of the preceding Child				
Alive	44	88.0	93	93.0
Dead	6	12.0	7	7.0
Last child pregnancy plan				
Yes	43	86.0	92	92.0
No	7	14.0	8	8.0
Ante natal care in preceding pregnancy				
No follow up	15	30.0	43	43.0
One follows up	12	24.0	19	19.0
Two follow up	8	16.0	14	14.0
Three follow up	9	18.0	13	13.0
Four and above	6	12.0	11	11.0
Contraceptive used before last pregnancy				
Yes	34	68.0	88	88.0
No	16	32.0	12	12.0
Type of contraceptive(*n* = 122)				
Depo-Provera	30	78.0	61	61.0
Implant	6	12.0	11	11.0
oral pills/condom/	5	10.0	9	9.0
Decision maker about F/P				
Self (Mother)	17	34.0	51	51.0
Both husband and wife	18	36.0	36	36.0
Husband only	15	30.0	13	13.0
Duration of Breast feeding				
≤ 24 months	10	20.0	29	29.0
13–23 months	31	62.0	64	64.0
≥ 24 months	9	18.0	7	7.0
Maternal attitude towards contraceptives				
Positives	44	88.0	90	90.0
Negatives	6	12.0	10	10.0
Paternal attitude towards contraceptives				
Positives	39	78.0	82	82.0
Negatives	11	22.0	18	18.0

### Why not participants using modern contraceptives

Sixteen (32.0%) of cases and 12 (12.0%) controls were not use any modern contraceptive at the time of interview. Participants list various reasons for currently not utilizing contraceptives. The reasons for not currently using contraceptives among cases were fear of change in breast milk content as the result of contraceptives, feeling of not exposed to the risk of pregnancy due to breast feeding, religion reason, due to side effect and partner refusal to use contraceptives. Among controls the reason of not utilization contraceptives were, they used amenorrhea and calendar methods.

### Determinant factors affecting short inter birth interval practice

First bi-variable logistic regression was conducted among variables with *p*-values of 0.2 or less were included in the multi-variable logistic regression. As can be depicted from multivariable backward stepwise logistic regression modern contraceptive utilization, number of alive children, breast fed, decision power of contraceptives utilization and mothers who have no antenatal care follow up in preceding pregnancy were found to be significantly associated with short birth interval.

The odds of having short birth interval was higher among mothers who did not use modern contraceptive method before getting pregnant for the last child (AOR = 6.29, 95% CI (1.95, 20.24)) than those who had history of using modern contraceptives. Participants who had ≤2 alive children were strongly significantly associated with short birth interval (AOR = 5.57, 95% CI (1.47, 21.13)).

Husband decision of maternal contraceptive utilization was significantly associated with short birth interval as compare to maternal decision (AOR = 2.69,95% CI (1.05,6.88)). Mothers who breast fed for the preceding child for less than 24 months were 3 times more likely to have short inter birth interval than their counter parts of mothers who breast fed for 24 months or more (AOR = 3.42, 95% CI (1.38, 8.46)). The odds of having short birth interval was higher for mothers who did not have history of antenatal care follow up in preceding pregnancy than those who had (AOR = 3.52, 95% CI (1.27, 9.75)) (Table 3).

**Table 3:** Bivariate and multivariable analysis of determinant factors among reproductive age women in Farta Woreda, South Gondar Zone, Ethiopia, 2019

Variables	Cases(*n* = 50) Number (%)	Controls(*n* = 100) Number (%)	COR (95% CI)	AOR (95% CI)	*P*-values
Educational level of mothers					
Unable to read and write	13 (26.0)	27 (27.0)	.43 (.14, 1.29)	1.08(.25, 4.65)	.909
able to read and write	18 (36.0)	19 (19.0)	.86 (.29, 2.51)	1.79(.43, 7.43)	.418
grade 1–12	8 (16.0)	45 (45.0)	.16 (.05, .51)	.35(.09, 1.32)	.123
college and above	11 (22.0)	9 (9.0)	1		
Maternal occupation					
house wife	19 (38.0)	57 (57.0)	.19 (.06, .52)	.19(.03, 1.10)	.064
farmer	17 (34.0)	35 (35.0)	.27 (.09,.78)	.44(.08, 2.43)	.352
merchant	14 (28.0)	8 (8.0)	1		
Age at fist pregnancy					
15–19	14 (28.0)	46 (46.0)	.30(.10, .91)	2.92 (.67, 12.76)	.153
20–24	27 (54.0)	44 (44.0)	.61(.21, 1.73)	.85(.23, 3.16)	.815
≥25	9 (18.0)	10 (10.0)	1		
Abortion before the last birth					
Yes	6 (12.0)	24 (24.0)	.43 (.164, 1.13)	.48(.13, 1.78)	.273
No	44 (88.0)	76 (76.0)	1		
Number of living children					
0–2	20 (40.0)	18 (18.0)	6.66 (2.27, 19.50)	5.57 (1.47, 21.13)	.**011**
3–4	24 (48.0)	46 (46.0)	3.13 (1.15, 8.46)	2.27(.70, 7.37)	.170
> =5	6 (12.0)	36 (36.0)	1		
Sex of the preceding child					
Female	21 (42.0)	56 (56.0)	.56 (.28, 1.13)	1.07(.43, 2.66)	.871
Male	29 (58.0)	44 (44.0)	1		
Status of the preceding Child					
Alive	44 (88.0)	92 (92.0)	1		
Dead	6 (12.0)	8 (16.0)	1.56 (.51, 4.79)	.61(.14, 2.52)	.498
Last pregnancy plan					
Yes	41 (82.0)	94 (94.0)	1		
No	9 (18.0)	6 (12.0)	3.43 (1.14, 10.29)	.529(.00, 68.97)	.798
ANC follow up in preceding pregnancy					
Yes	35 (70.0)	57 (57.0)	1		
No	15 (30.0)	43 (43.0)	.56 (.27, 1.17)	3.52 (1.27, 9.75)	.**015**
Decision maker about F/P					
Self (Mother)	17 (34.0)	51 (51.0)	1		
Both husband and wife	18 (36.0)	36 (36.0)	1.50(.68,3.29)	1.40(.60, 3.24)	.431
Husband only	15 (30.0)	13 (13.0)	3.46 (1.37,8.71)	2.69 (1.05,6.88)	**.038**
Maternal attitude towards family planning					
Positives	44 (88.0)	90 (90.0)	1		
Negatives	6 (12.0)	10 (10.0)	1.22 (.41, 3.59)	1.30 (.34, 4.99)	.696
Paternal attitude towards family planning					
Positives	38 (76.0)	83 (83.0)	1		
Negatives	12 (24.0)	17 (17.0)	1.54 (.67, 3.54)	.85(.23, 3.12)	.814
Contraceptive use					
Yes	31 (62.0).0	91 (91.0)	1		
No	19 (38.0)	9 (9.0)	6.19 (2.54, 15.11)	6.29 (1.95, 20.24)	.**002**
Duration of Breast feeding					
< 24 months	34 (68.0)	32 (32.0)	1		
24 months	16 (32.0)	68 (68.0)	4.51 (2.18, 9.34)	3.42 (1.38, 8.46)	.**008**

## Discussion

This study was aimed to identify factors influencing short inter birth interval among child bearing age mothers who had at least two live births with their last child born within the six months in rural Farta district south Gondar Ethiopia. Short inter birth interval is critical in sub Saharan African countries including Ethiopia, where perinatal mortality and fertility remains still high. However, birth spacing is affected by different factors such as modern contraceptive utilization, number of alive children, breast fed, decision of contraceptives utilization and mothers who have no antenatal care follow up were found to be significantly associated with short birth interval.

Contraceptive utilization before gating last pregnancy was found to be a predictor of short birth interval. The finding of this study indicated that mothers who not utilized modern contraceptive method before they got pregnant for her last child were more likely to experience short birth interval than mothers who used contraceptive methods. In line with evidences from studies done in rural pastoral communities of southern Ethiopia, Mekele, Lemo district and Arbaminch district, Ethiopia [12, 17, 18, 21] were associated factors, respectively. This might be due to married women are at greater risk for contraceptive methods nonuse; this causes unintended conceptions which lead to affect birth interval. Participants reason for non-use of contraceptives, were fear of change in breast milk content, feeling of not exposed to the risk of pregnancy due to breast feeding, religion reason, side effect and partner refusal to use contraceptives.

Mothers who had less than or equal to two number of alive children were significantly associated with short birth interval. This might be due to women want many numbers of children and number of children are considered as asset especially in rural resident parents.

Mothers who breast fed the preceding child for less than 24 months was more likely to have short inter birth interval than their counter parts of mothers who breast fed for 24 months and above. This study finding was similar with evidences from southern Ethiopia, Manipur, Nigeria, Mekele and Arbaminch district [12, 15–17, 21]. As opposed to our finding, study from Lemo District, showed that duration of breast-feeding had inverse relationship with birth intervals [18]. This might be due to the fact that breast feeding extends period of inter birth interval through negative hormonal feedback. Breastfeeding leads to secretion of prolactin hormone from the pituitary gland and lower follicular secretion hormone and luteinizing hormone levels in the blood. Subsequently, ovulation is delayed and the amenorrhea period prolongs, reducing the risk of fertility [22].

Husband who had decision power on maternal contraceptive utilization was another determinant factor of birth interval as compared to maternal her own decision on contraceptive utilization. This was supported by study done in Nepal and rural area of western Ethiopia [23, 24], respectively. This might be as a result of women decision-making ability or autonomecity was vital in selecting contraceptive types to prevent closely birth spacing and went to the health institution for family planning service. Autonomous mothers might defend myths and bypass everything which affect their right of attaining quality health care service especially gaining charge free maternal care service.

The likelihood of having short birth interval was 3 times higher for mothers who did not attend antenatal care follow-up during the preceding pregnancy as compared to those who attended it. This might be due to lack of information about family planning counseling and health education during antenatal care follow up and lack of health facility may deter women adhering to optimal birth spacing.

### Limitation

Memory bias might be introduced and this study focus on Quantitative approach.

Which could not address the “why” questions in detail.

## Conclusion and recommendation

Optimal birth interval has significant role in reducing fertility and maternal and child morbidity and mortality. Variables affecting birth interval were modifiable factors. Thus, creating awareness the effect of short birth interval for maternal and children health and impact of high fertility on country, health information about benefit of breast feeding, antenatal care follows up during pregnancy, contraceptive utilization after delivery and empower decision making ability of mothers on their own health and contraceptive utilization to optimize birth spacing for rural communities.

## Data Availability

The data sets generated during this study are available from the correspondence author on reasonable request via email.

## Abbreviations

ANC: Ante Natal Care
EDHS: Ethiopian demography and health survey
SD: Standard deviation
